# Study on the temporal and spatial distribution of *Culex* mosquitoes in Hanoi, Vietnam

**DOI:** 10.1038/s41598-024-67438-3

**Published:** 2024-07-17

**Authors:** Janina Krambrich, Thang Nguyen-Tien, Long Pham-Thanh, Sinh Dang-Xuan, Ella Andersson, Patrick Höller, Duoc Trong Vu, Son Hai Tran, Lieu Thi Vu, Dario Akaberi, Jiaxin Ling, John H.-O. Pettersson, Jenny C. Hesson, Johanna F. Lindahl, Åke Lundkvist

**Affiliations:** 1 Department of Medical Biochemistry and Microbiology, Zoonosis Science Center (ZSC), Uppsala University, Uppsala, Sweden; 2 International Livestock Research Institute, Hanoi, Vietnam; 3 Department of Animal Health, Ministry of Agriculture and Rural Development (MARD), Hanoi, Vietnam; 4 National Institute of Hygiene and Epidemiology, Hanoi, Vietnam; 5 Biologisk Myggkontroll, Nedre Dalälvens Utvecklings AB, Gysinge, Sweden; 6 Department of Animal Health and Antibiotic Strategies, Swedish Veterinary Agency, Uppsala, Sweden; 7 Clinical Microbiology, Department of Medical Sciences, University of Uppsala, Uppsala, Sweden; 8 Clinical Microbiology and Hospital Hygiene, Uppsala University Hospital, Uppsala, Sweden; 9 Department of Microbiology and Immunology, Peter Doherty Institute for Infection and Immunity, University of Melbourne, Melbourne, VIC Australia

**Keywords:** Ecology, Viral vectors, Viral epidemiology

## Abstract

Arboviruses transmitted by mosquitoes, including Japanese encephalitis virus (JEV), present a substantial global health threat. JEV is transmitted by mosquitoes in the genus *Culex*, which are common in both urban and rural areas in Vietnam. In 2020, we conducted a 1-year survey of *Culex* mosquito abundance in urban, suburban, and peri-urban areas of Hanoi using CDC-light traps. Mosquitoes were identified to species and sorted into pools based on species, sex, and trap location. The mosquito pools were also investigated by RT-qPCR for detection of JEV. In total, 4829 mosquitoes were collected over a total of 455 trap-nights, across 13 months. Collected mosquitoes included *Culex*, *Aedes*, *Anopheles*, and *Mansonia* species. *Culex* mosquitoes, primarily *Cx. quinquefasciatus*, predominated, especially in peri-urban areas. Most *Culex* mosquitoes were caught in the early months of the year. The distribution and abundance of mosquitoes exhibited variations across urban, suburban, and peri-urban sites, emphasizing the influence of environmental factors such as degree of urbanization, temperature and humidity on *Culex* abundance. No JEV was detected in the mosquito pools. This study establishes baseline knowledge of *Culex* abundance and temporal variation, which is crucial for understanding the potential for JEV transmission in Hanoi.

## Introduction

Mosquitoes are key players in the spread of vector-borne diseases, such as Japanese encephalitis and West Nile fever, representing a significant global health concern. Vector-borne viruses collectively account for close to 700,000 deaths each year and are rapidly emerging as prominent threats to local populations^1,2^. Factors that may change vector dynamics and can alter vector abundance, such as urbanization, changes in land-use, deforestation, and climate change contribute to the escalating prevalence of mosquito-borne illness worldwide^3^.Understandng the abundance and dynamics of mosquito vectors is crucial for assessing the risks of arboviral diseases and anticipating future outbreaks^4–6^, and to enable timely implementation of control measures to mitigate the risk of human infection^4^.

Mosquito-borne diseases are highly prevalent in South-East Asia, including Vietnam^3^, and Japanese Encephalitis virus (JEV), dengue virus (DENV), Zika virus (ZIKV), and chikungunya virus (CHIKV) are examples of viruses endemic in Vietnam^2,3^.JEV is a highly pathogenic mosquito-borne flavivirus, that causes encephalitis in humans and horses in tropical and temperate regions of Asia^7,8^. Pigs act as amplifying hosts and can be affected by the virus, mainly resulting in abortions and neonatal deaths^7,8^. The primary vectors responsible for JEV transmission are *Culex* mosquitoes, particularly *Cx. tritaeniorhynchus*, *Cx. vishnui*, and *Cx. gelidus*. *Cx. quinquefasciatus* sometimes act as an additional vector for JEV^9–11^. Occasionally, other mosquito genera are also reported to carry the virus^12–14^.

A combination of increased urbanization and the rapidly expanding agricultural sector across Southeast Asia has contributed to the higher risk of JE infection, as people move closer to rice fields and livestock, where there is an increase in JEV vector mosquitoes and amplifying hosts^15^. The surge in meat production, particularly pork, throughout Asia, has exponentially escalated the number of amplifying hosts the past decades^13^. Furthermore, the rapid urbanization and expansion of peri-urban livestock rearing, especially urban pig farming, have brought amplifying hosts closer to urban, more densely populated areas^13,16^. Climate change and extreme weather phenomena, such as floods, have likely facilitated the expansion of vector mosquitoes into previously untouched areas, including highland regions, and closer to urban areas^16^. While mosquitoes are active year-round in tropical areas, their activity in temperate zones may peak during specific seasons when conditions, such as warmer temperatures and increased precipitation, are favorable for mosquito breeding^17^.

Despite available JEV vaccines and vaccination campaigns, the virus remains endemic in 24 countries in South and Southeast Asia, where approximately three billion people are at risk of infection^18,19^. This study is motivated by the significant disease burden posed by JEV, and aims at increasing our knowledge of urban JEV vector population dynamics in Hanoi, Vietnam. By collecting mosquitoes in three different environments throughout a whole year, we contribute to a comprehensive understanding of fluctuations in *Culex* mosquito abundance, which is critical for implementation of effective JEV disease control and prevention. Additionally, collected vector species were screened for the presence of JEV.

## Methods

### Field sampling and speciation

**Figure 1:**
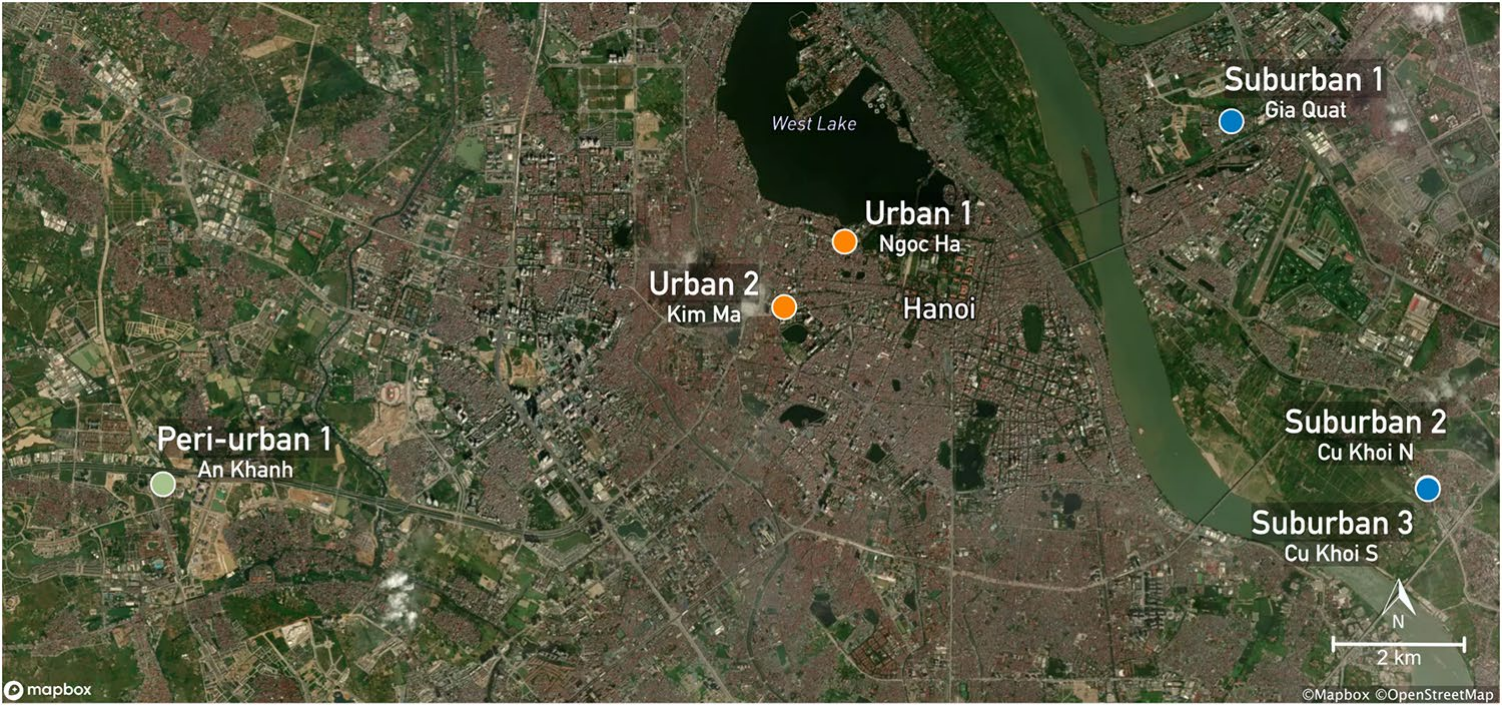
Location of CDC Light Traps for Mosquito Collection. The CDC light traps were strategically placed in different locations to capture mosquito populations from diverse settings. The traps were positioned in An Khanh (Peri-urban 1), representing peri-urban Hanoi (green); Gia Quat (Suburban 1) and Cu Khoi (Suburban 2 and 3) (blue), representing suburban areas; and Ngoc Ha (Urban 1) and Kim Ma (Urban 2) (orange), representing urban Hanoi. Two traps were placed in adjacent houses in Cu Khoi, one to the north (N) and one to the south (S). The map is based on OpenStreetMap (https://www.openstreetmap.org/about/) data, and the locators were added using Mapbox GL (https://www.mapbox.com/about/maps).

Mosquitoes were collected in Hanoi, Vietnam, using CDC miniature light traps (Bioquip), with incandescent bulbs and motorized fans, placed at five sites (Fig. 1). CDC miniature light traps were employed as they have shown to be effective in collecting *Culex* mosquitoes, the main JEV vector genus^20,21^. Due to local restrictions, dry ice was not available as a CO_2_ bait. The selected sites included three different environments, including urban (Ngoc Ha, Kim Ma), suburban (Gia Quat, Cu Khoi), and peri-urban (An Khanh) areas, that were approximately 10–20 km apart from each other. Geo-coordinates are provided in Supplementary Table S1. Two traps were placed in different households in the same suburban neighbourhood in Cu Khoi. Urban areas were characterized by high population density (~ 24,000 people/km^2^), non-agricultural environments, and numerous human structures, while peri-urban areas had lower population density (~ 3000 people/km^2^), agricultural landscapes including rice fields and grasslands, and various agricultural water sources. Suburban regions had a semi-dense population (~ 5400 people/km^2^) with parks and green spaces, but no agriculture, along with a moderate number of small, isolated water basins. Individual traps were repeatedly placed in the same location. One trap per site and trapping night was positioned outside human settlements (in the garden or on the terrasse), 150 cm above the ground, and were activated twice weekly (Monday and Thursday) from 6 pm to 7 am the next morning, over a thirteen-month period, from January 2020 to January 2021. Daily catches were stored at − 20 °C immediately after collection and brought to − 80 °C within one month for preservation until identification and sorting. Collected mosquitoes were identified to species at the National Institute of Hygiene and Epidemiology (NIHE), under the Ministry of Health of Vietnam, using the morphological key by Stojanovich and Scott and stored at − 80 °C until further analysis. In addition, temperature and humidity were recorded with analogue thermo- and hygrometers at each trap site at each sampling occasion.

### Extraction and RT-qPCR

The frozen mosquito samples were transported by air on dry ice from Vietnam to the Zoonosis Science Centre (ZSC) at Uppsala University, Sweden. The mosquitoes were then pooled, each pool containing a maximum of 50 individuals, based on species, sex, trap location, and collected within two consecutive weeks of each other (Table 1). A detailed pooling scheme can be found in Supplementary Table S2. Mosquito homogenization and inactivation of potentially infectious viruses were conducted at biosafety level (BSL) 3, and downstream applications was performed at BSL2. Homogenization was achieved through bead beating the mosquito pools in phosphate buffered saline (PBS) (Gibco™, Thermo Fisher Scientific, Inc.) with 10% fetal bovine serum (FBS) (Gibco™, Thermo Fisher Scientific, Inc.), 1% Penicillin–Streptomycin (PenStrep) (Gibco™, Thermo Fisher Scientific, Inc.), and 0.1% Amphotericin B (AmphB) (Gibco™, Thermo Fisher Scientific, Inc.). Five hundred microliter of buffer were added for pools containing < 15 mosquitoes, 750 µL for pools with 15–30 mosquitoes, and 1000 µL for pools containing 30–50 mosquitoes. Samples were homogenized using 5 mm stainless steel beads in the Minilys® personal homogenizer (Bertin Corp., MD, USA) bead mill set to a frequency of 25 Hz for 2 min.

**Table 1:** Number of pools containing the different mosquito species, sex (F = female mosquitoes, M = male mosquitoes), and number of mosquitoes.

Species	Sex	Pools	Mosquitoes
*Cx. quinquefasciatus*	F	156	3701
*Cx. quinquefasciatus*	M	109	665
*Cx. tritaeniorhynchus*	F	38	185
*Ae. albopictus*	M	20	26
*Ae. albopictus*	F	14	19
*Cx. gelidus*	F	13	108
*Cx. vishnui*	F	3	6
*Ma. annulifera*	F	3	4
*Ma. uniformis*	F	3	4
*Cx. gelidus*	M	2	3
*Armigeres*	M	2	2
*Cx. pseudovishnui*	F	1	1
*Ae. vexans*	F	1	1
*Ae. aegypti*	F	1	2
*Ma. indiana*	F	1	1
*Armigeres*	F	1	1
*Cx. tritaeniorhynchus*	M	1	3
*Cx. vishnui*	M	1	1
*Ae. aegypti*	M	1	1
Total		371	4734

Viral RNA extraction was performed using the QIAampⓇ Viral RNA Mini Kit (Qiagen, Hilden, Germany) following the manufacturer's instructions. Elution was conducted in water, and RNA samples were temporarily stored at − 20 °C until RT-qPCR analysis, after which they were transferred to − 80 °C for long-term storage. For the detection of viral RNA, a one-step quantitative reverse transcription PCR (RT-qPCR) assay were employed.

Inhouse designed JEV primers were designed: forward 5′-GGCTAGCCTACAAGGTGGCG-3′ and reverse 5′-CTCTCGCCCATTCGGGTGAC-3′^22^. They amplify a 110 bp long sequence in the NS5 gene region of the viral genome. The detection limit of the assay was determined to be between one and 10 copies/µl.

The RT-qPCR assays were conducted using the QuantiTect® SYBR® Green RT-PCR kit (Qiagen, Hilden, Germany). The PCR reaction mix contained 12.5 μL 2 × QuantiTect SYBR RT-PCR Master Mix (HotStarTaq® DNA Polymerase, QuantiTect SYBR RT-PCR Buffer, dNTP mix, including dUTP, ROX™ passive reference dye, 8 mM MgCl_2_, and SYBR Green I dye), RNAse-free water, 10 µM forward primer, 10 µM reverse primer, and 0.25 μL QuantiTect RT Mix (Omniscript® Reverse Transcriptase and Sensiscript® Reverse Transcriptase) per reaction. A total of 20 μL master mix was pipetted in each reaction well and 5 µL extracted RNA were used as template in all reactions. The PCRs were run using the CFX Connect Real-Time PCR Detection System (Bio-Rad, Hercules, California, USA). PCRs were run with 30 min reverse transcription (RT) at 50 °C, 15 min initial activation at 95 °C, followed by 45 cycles of 15 s at 94 °C, 30 s at 58 °C, and 30 s at 72 °C, followed by a melting curve. All amplicons giving a signal were used for gel electrophoresis. As positive control a synthetic DNA gene fragment (gBLOCKs; IDT®, San Jose, CA, USA) with three-base pair insertion in the corresponding amplified regions of the viral genome diluted in deionized, nuclease-free water were used.

### Case data

Cases of viral encephalitis in the northern Vietnam region (Đông Bắc Bộ, Tây Bắc Bộ, and Đồng Bằng Sông Hồng) were obtained from the weekly announcements on the infectious disease epidemic in the northern region by NIHE. JE cases are not reported separately since diagnostics are not always available, however, according to NIHE, JEV is the primary cause of acute encephalitis syndrome (AES) in rural plains and mountainous areas of Vietnam. Additionally, viruses such as intestinal virus ECHO 30, Banna virus, Nam Dinh virus, and herpes virus are identified as causes of AES in Vietnam^23^.

### Data analysis

To identify significant differences (**** = *p* < 0.0001, *** = *p* < 0.001, ** = *p* < 0.01, * = *p* < 0.05, ns = not significant) in mosquito counts, Chi-square and ANOVA tests were performed using GraphPad Prism (version 10.0.0 for MacOS, GraphPad Software, Boston, Massachusetts USA, www.graphpad.com). The map for visualising the locations for mosquito trapping was produced using mapbox (v. 3.1.2) and OpenStreetMap.

## Results

### Mosquitoes, temperature, and humidity

**Figure 2:**
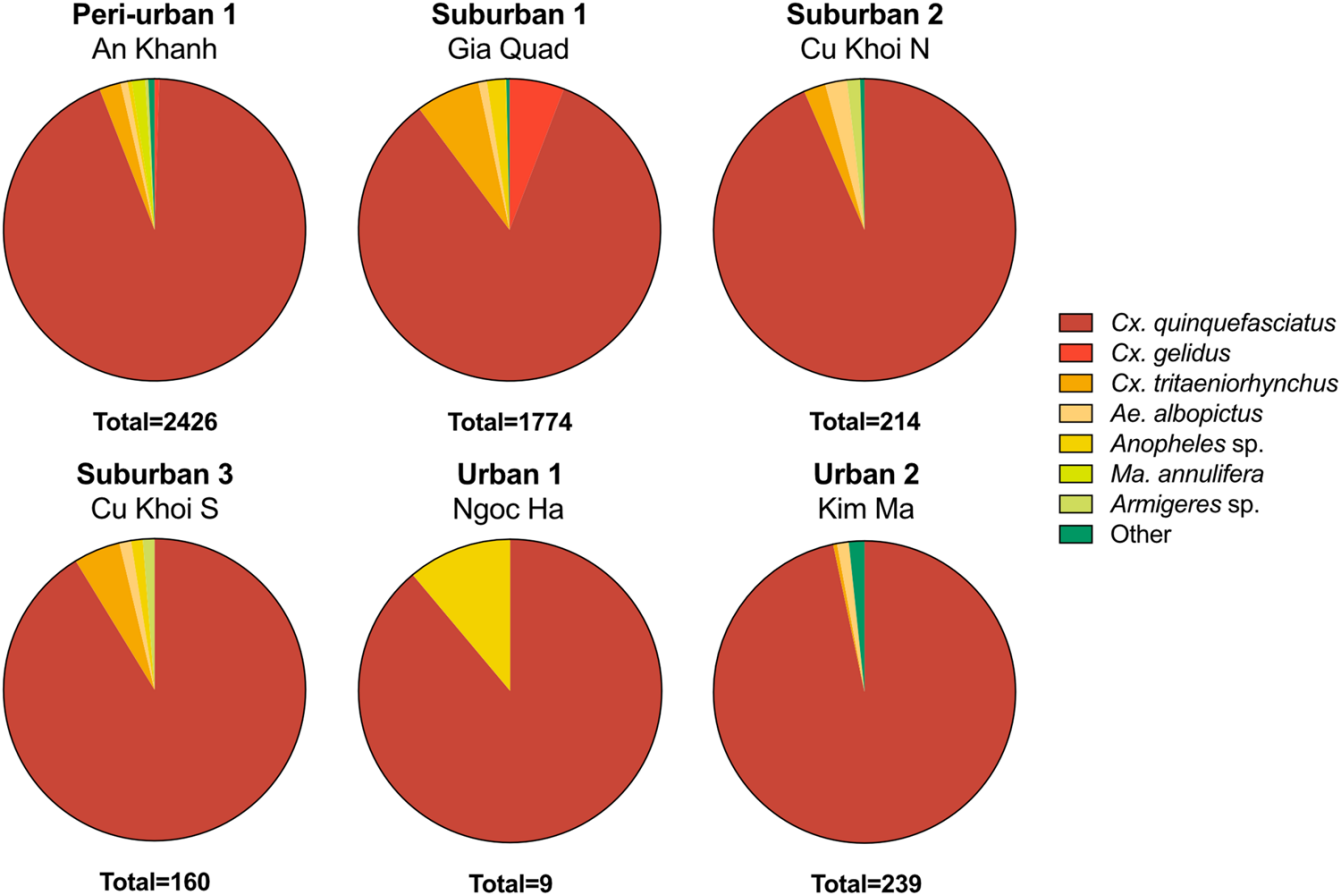
Mosquitoes collected over a 13-month period, sorted by species and trap. Species with over ten specimens collected throughout the traps are specified, species with less than ten specimens in all traps are summarized as “Other”. “Other” comprises *Cx. pseudovishnui, Cx. vishnui, Ae. vexans, Ae. aegypti, Ma. indiana, Ma. uniformis, Ma.* spp*.* and* Armigeres kuchingensis.*

**Figure 3:**
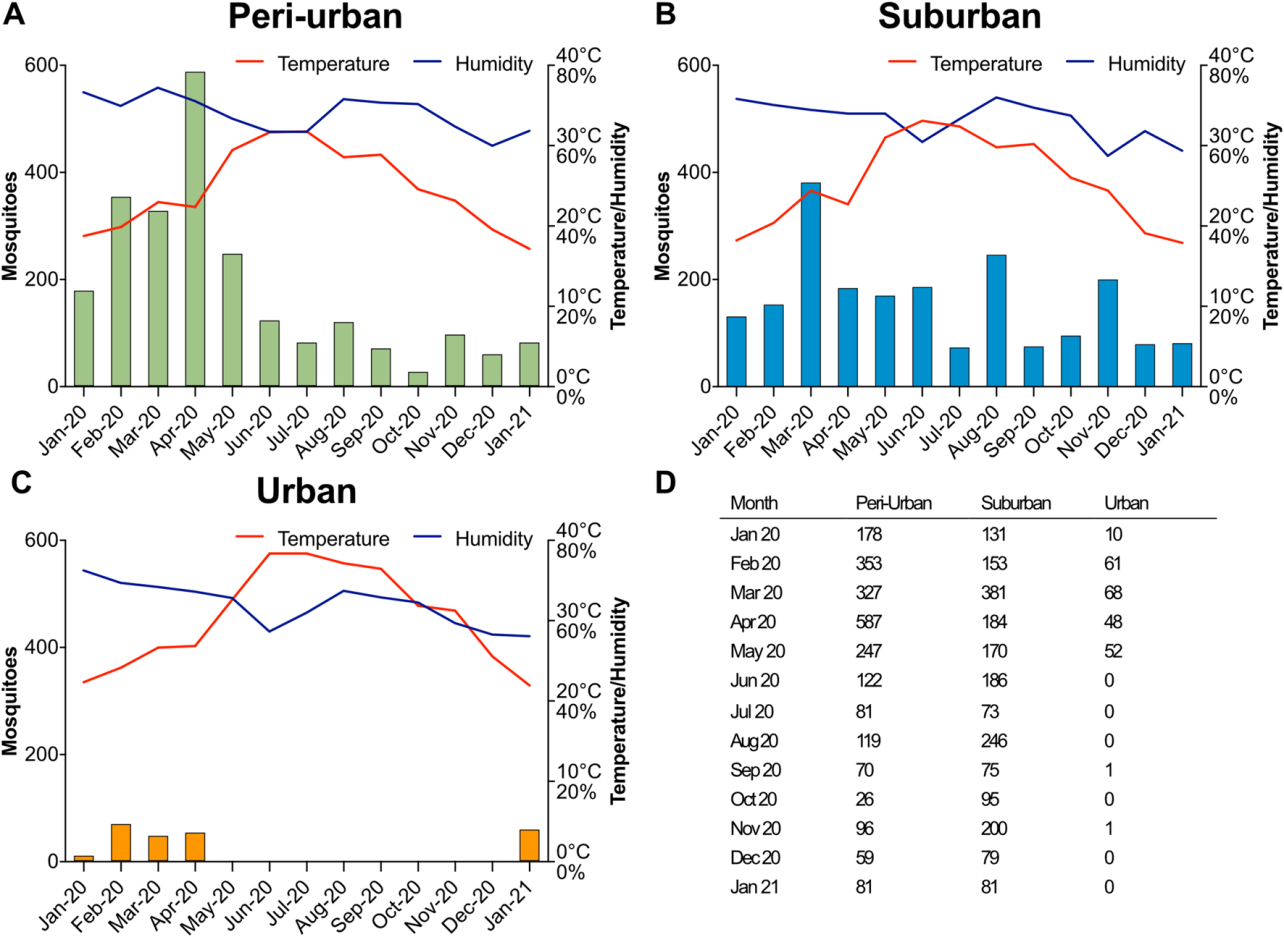
Seasonal variation in *Culex* mosquitos, temperature, and humidity in three different areas. The graphs display the number of *Culex* mosquitoes collected, and the average monthly temperature and humidity recorded at the (**A**) peri-urban, (**B**) suburban, and (**C**) urban trapping sites in the different months throughout the year. The table in (**D**) summarizes the *Culex* mosquito counts.

A total of 4829 mosquitoes were collected from six traps over a 13-month period, for a total of 106 ± 1 trap nights per site for five traps, and for 33 nights for the sixth trap (Urban 2—Kim Ma) that had to be discontinued due to government regulations during the COVID-19 pandemic. The species distribution is presented in Fig. 2 and in Supplementary Table S3. Among the collected mosquitoes, the genus *Culex* dominated, accounting for 96.5% of the total count. Specifically, *Cx. quinquefasciatus* was the most abundant species, followed by *Cx. tritaeniorhynchus*, *Cx. gelidus*, *Cx. vishnui*, and *Cx. pseudovishnui*. Mosquitoes belonging to the genera *Aedes*, *Anopheles*, and *Mansonia* each comprised 1% of the collected specimens, while mosquitoes from the genera *Armigeres* and *Ficalbia* constituted 0.45% and 0.04%, respectively, of the total collection (Fig. 2, Supplementary Table S3). Throughout the year, we collected 4641 *Culex*, 49 *Aedes*, 22 *Armigeres*, and 49 *Anopheles* mosquitoes in varying quantities over the months (Supplementary Figure S1). Additionally, 46 *Mansonia* mosquitoes were collected, but only between August through November in the Peri-urban 1 and Suburban 1 traps (Supplementary Figure S1). The *Culex* count, and the total mosquito count were similar in the Peri-urban 1 and the combined Suburban traps (Fig. 3, Supplementary Figure S1). Overall, the Peri-urban 1 trap recorded the highest number of total mosquitoes caught, followed by the Suburban trap 1, then the other two Suburban traps. In the Urban 2 trap, numbers were similar to the Suburban 2 and 3 traps before trapping was discontinued. Urban 1 recorded the lowest number of mosquitoes over time.

Throughout the year, we collected 4641 *Culex* mosquitoes. In the two Urban traps, 241 *Culex* mosquitoes were collected over the year (Fig. 3C). In the three Suburban traps a total of 2054 mosquitoes were caught (Fig. 3B) and a total of 2346 mosquitoes were collected in the Peri-urban trap (Fig. 3A, Supplementary Table S3). Considering that we had three Suburban traps, partially two Urban traps and one Peri-urban trap, the average number of *Culex* mosquitoes per trap and area were compared. There was no significant difference between the amount of caught *Culex* mosquitoes in the Urban and Suburban traps (*p* = 0.4742), but the Peri-urban trap captured significantly more *Culex* mosquitoes than both the Suburban (*p* = 0.0031) and Urban (*p* = 0.0002) traps on average (Supplementary Table S3).

*Culex* mosquitoes showed a peak in abundance during the early months of the year, from February to May, with fewer mosquitoes collected during the rest of the year (Fig. 3).

The average monthly temperature was significantly higher in the Urban traps when compared to the Suburban (*p* < 0.0001) and Peri-urban (*p* < 0.0001) traps respectively (Fig. 3, Supplementary Table S4). There was no significant difference in average monthly temperature between the Suburban and Peri-urban traps throughout the year (*p* = 0.1063) (Fig. 3, Supplementary Table S4). The summer and early autumn months (May to September) were significantly warmer compared to the rest of the year (*p* < 0.0001) (Fig. 3, Supplementary Table S4).

The average monthly humidity was significantly lower in the Urban traps when compared to the Suburban (*p* = 0.0089) and Peri-urban (*p* < 0.0001) traps respectively. There was no significant difference in average monthly humidity between the Suburban and Peri-urban traps (*p* = 0.2166) (Fig. 3, Supplementary Table S4). June and July were on average significantly more humid (*p* < 0.0001), whereas November, December and January were significantly less humid (*p* < 0.0001), compared to the other months (Fig. 3, Supplementary Table S4).

### Seasonal variation in viral encephalitis cases

**Figure 4:**
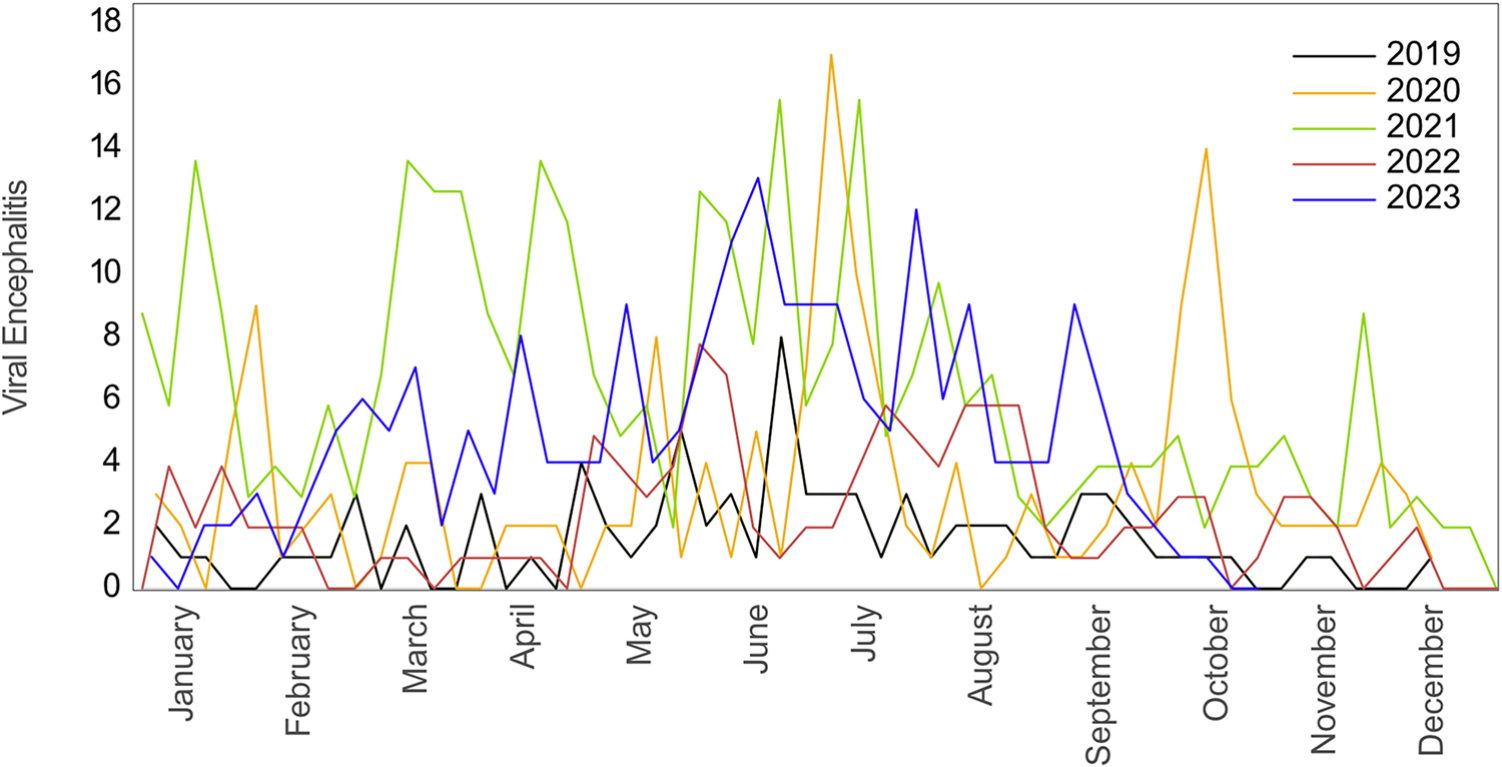
Reported cases of viral encephalitis in the northern Vietnam region between 2019 and 2023. The figure is based on reported cases of disease by the National Institute of Hygiene and Epidemiology, Vietnam. Viruses such as JEV, intestinal virus ECHO 30, Banna virus, Nam Dinh virus, and herpes virus could cause viral encephalitis, but JEV is the most common causative agent in northern Vietnam.

Cases of viral encephalitis in the northern Vietnam region were reported by NIHE. In total 1478 cases of viral encephalitis were reported between 2019 and 2023 (329 [2019], 468 [2020], 344 [2021], 123 [2022], 214 [2023]). Cases do not show clear seasonality (Fig. 4). In 2018 and 2023 however, more cases were recorded in the summer months. In 2019, 2020, 2021, and 2022 a similar number of cases were reported throughout the year (Fig. 4). Considering the population of 36,931,761 [2019] inhabitants in northern Vietnam^24^, this results in an incidence of 0.9 [2019], 1.3 [2020], 0.9 [2021], 0.3 [2022], 0.6 [2023] per 100,000 per year.

### Screening of viral RNA in vector mosquito pools

Out of the total 4829 collected mosquitoes, 4673 were from the genus *Culex* and therefore potential JEV vector species (Table 1). In total 324 pools with *Culex* mosquitoes were analysed and JEV viral RNA could not be detected in any of the pools.

## Discussion

In this study, *Culex* mosquitoes were collected throughout a whole year in Hanoi, Vietnam, to investigate their seasonality and distribution at different levels of urbanisation. In total, 4829 mosquitoes over collected over 13 months from six traps, with *Culex* mosquitoes dominating the catch (96.5%), particularly *Cx. quinquefasciatus* with peak abundance from February to May.

Previous studies have identified *Culex* mosquitoes as important vectors for JEV^9–14^. *Cx. tritaeniorhynchus* is the primary vector of JEV in most endemic areas^13,25^. It is closely associated with rice cultivation and wetlands, which are ideal breeding sites for this mosquito species^13,25^. In the current study, *Cx. tritaeniorhynchus* was the second most collected species. Instead, *Cx. quinquefasciatus* vastly dominated the catch throughout the year at all sites, which is not uncommon in urban areas ^26–28^.

This species breeds in a variety of artificial and natural water bodies and is commonly found in both urban and rural environments^5^. In this study, most mosquitoes were collected in the peri-urban area followed by the suburban area, and the fewest mosquitoes were collected in the urban area. This is consistent with another study conducted in September and October 2018 in the same area around Hanoi, which also found that there were fewer mosquitoes in urban areas than in peri-urban areas^21^. This could be due to higher awareness in urbanized settings, leading to better implementation of mosquito control, or less availability of breeding sites and blood-meal hosts^3^. Additionally, we found that the average monthly temperatures in the urban area were higher and the average monthly humidity was lower when compared to both the suburban and peri-urban areas. Lower humidity and higher temperatures shorten mosquito lifespan and could lead to reduced reproduction resulting in fewer mosquitoes^29,30^. In addition, the different trapping results between urban and peri-urban areas suggest that urbanization and habitat characteristics, as shown previously, could also play an important role in mosquito abundance and distribution patterns^31–33^.

*Cx. quinquefasciatus* is able to transmit JEV in laboratory experiments^34,35^, and is also an important vector of West Nile virus^36^, and it is the main vector of Bancroftian lymphatic filariasis in tropical and subtropical regions. It commonly feeds from mammals, including humans. A blood-meal analysis study found that 93.3% of Mexico *Cx. quinquefasciatus* had consumed mammalian blood, with human blood accounting for the majority at 65.4%. Other sources included dog (23.2%), chicken (5.4%), goat (2.2%), and cat (1.8%) blood^37^. In Benin, the vast majority had also fed on humans (88.5%), with the rest feeding on goats (6.5%), cattle (3%), and pigs (2%)^38^.

JEV has previously been isolated from several *Culex* species in the field. In central and southern Thailand, the minimum infection rate (MIR) per 1000 collected mosquitoes was 2.0 for JEV in *Cx. quinquefasciatus* and in West Bengal, India, the MIR was 2.5^39,40^. In Vietnam JEV has been isolated from *Cx. tritaeniorhynchus* mosquitoes between 2006 and 2008^26^. In this study, we also analysed the collected mosquitoes for JEV. With the limited number of mosquitoes collected (4673 *Culex* mosquitoes, 4001 females), detection of JEV was unlikely but possible, as the median field infection rate in *Culex* species from other countries in Southeast Asia, China, and Japan was 0.71 mosquitoes per 1000 specimens tested^26,27,39–49^. At a similar field infection rate, we would have expected 2.8 infected female mosquitoes in our collected material. However, reported infection rates vary between 0.05 and 5.29, depending on the country, season, and site of collection, and in a study conducted in 2002 and 2004 in northern Vietnam, JEV was also not detected in the 20,615 mosquitoes tested^50^.

Seasonal variations in field infection rates as well as in vector abundance are important to consider when implementing mosquito surveillance and control strategies. Our study shows peak activity of *Culex* mosquitoes in the early months of the year, suggesting control would be best implemented before and during observed peak season. Similar patterns of abundance have been observed in other studies. Studies investigating the seasonal abundance of *Culex* mosquitoes in Southeast Asia found, similar to our results, that these mosquitoes mainly occur in the early months of the year through late summer, with a dip in the fall. In 2009, Ohba et al. caught ten times as many *Culex* mosquitoes in Hanoi in July compared to October, but did not sample in spring^51^, and in 2017, Boyer et al. collected most *Culex* mosquitoes in Cambodia in June, followed by December and March, with the fewest mosquitoes collected in September^52^. Soh et al.^53^ found that from 2009 to 2018, *Culex* larva in Singapore were mainly found between February and June, and in 2010 Zhang et al.^54^ found that *Culex* adults in China`s Yunnan Province were most abundant between March and August. Thus, the timing of peak abundance of *Culex* may differ slightly between years, perhaps due to regional differences in climate or due to annual fluctuations in weather conditions. Soh et al.^53^ found in their study, that larval activity increased at higher temperatures and decreased at higher rainfall, suggesting that intra-annual climate variability may play a role in shifting the window of highest mosquito abundance. Our study year 2020 was generally a warmer year than usual, with increased precipitation in the winter and spring and dryer summer when compared to the mean temperatures and precipitation in Hanoi between 1979 and 2023^55^. Meteoblue, a meteorological service created at the University of Basel, Switzerland, reports increased temperatures and decreased precipitation in Hanoi over the past 50 years^55^. These trends could change the mosquito population dynamics further and additional surveillance will be needed to monitor the effects on vector abundance.

The reported annual incidence of AES in Vietnam ranged from 3.0 to 1.4 cases per 100,000 population between 1998 and 2007, and the national monthly incidence of AES declined by 63.3% between 1998 and 2016^56–58^. The incidence of AES however increased in some provinces, particularly in the Northwest region, and it has been reported that the incidence peaked in the summer months in northern Vietnam, in contrast to the southern provinces where incidence remained relatively constant throughout the year^58^. This correlates to the higher number of *Culex* mosquitoes in the earlier summer months as reported here and in other studies^51,52^. In our study, the viral encephalitis incidence in the northern Vietnam region ranged from 0.6 to 1.3, which would indicate a further decline when compared to earlier reported cases of AES^56–58^. Laboratory testing, however, is not consistently available to differentiate JE from other causes, so viral encephalitis cases are counted and reported as a surrogate for JE surveillance.

## Conclusion

Our study contributes to the understanding of *Culex* mosquito abundance in Hanoi, Vietnam. We show that *Cx. quinquefasciatus* dominated the collections and peaked in abundance from February to May, and was more common in the peri-urban area, which were characterized by being less densely populated with an increase in green spaces, gardens, and urban farms. Our findings provide vital baseline data on *Culex* mosquito abundance throughout a whole year in a large metropolitan area in Southeast Asia. Additionally, we highlight the need for ongoing surveillance efforts, to better comprehend the interplay between mosquitoes and arboviruses. Ultimately, this knowledge will be crucial in formulating targeted interventions and strategies to mitigate the impact of vector-borne diseases on public health.

### Supplementary Information

Supplementary Information 1.

Supplementary Information 2.

Supplementary Information 3.

Supplementary Information 4.

Supplementary Information 5.

Supplementary Information 6.

## Data Availability

All data generated or analyzed during this study are included in this published article.
