# Supplementary material for: Study on the temporal and spatial distribution of Culex mosquitoes in Hanoi, Vietnam
Source: Sci Rep. 2024 Jul 17;14:16573. doi: 10.1038/s41598-024-67438-3 (PMC11255287; doi:10.1038/s41598-024-67438-3)
Supplement: Supplementary file 1 — Supplementary Information 1. [file 41598_2024_67438_MOESM1_ESM.docx]

# Supporting information

**S1 Figure. Seasonal variation in *Aedes, Anopheles, Mansonia,* and *Armigeres* mosquitos, temperature, and humidity in three different areas.** The graphs display the number of *Aedes, Anopheles, Mansonia,* and *Armigeres* mosquitoes caught, and the average monthly temperature and humidity recorded at the (A) peri-urban, (B) suburban, and (C) urban trapping sites in the different months throughout the year. The table in (D) summarizes the mosquito counts.

**S1 Table. Geolocations of each trap.**

**S2 Table. Detailed pooling scheme.**

**S3 Table. Species composition.** Species composition of mosquitoes collected in the different traps in Hanoi during the sampling year 2020.

**S4 Table. Monthly average temperature and humidity recorded at each trap.** Temperature and humidity measurements were taken during both nightly trap placement and morning trap collection. Daily data was averaged per trap, and subseqeuntly per area and month.
