# Supplementary figures and images for: Study on the temporal and spatial distribution of Culex mosquitoes in Hanoi, Vietnam
Source: Sci Rep. 2024 Jul 17;14:16573. doi: 10.1038/s41598-024-67438-3 (PMC11255287; doi:10.1038/s41598-024-67438-3)

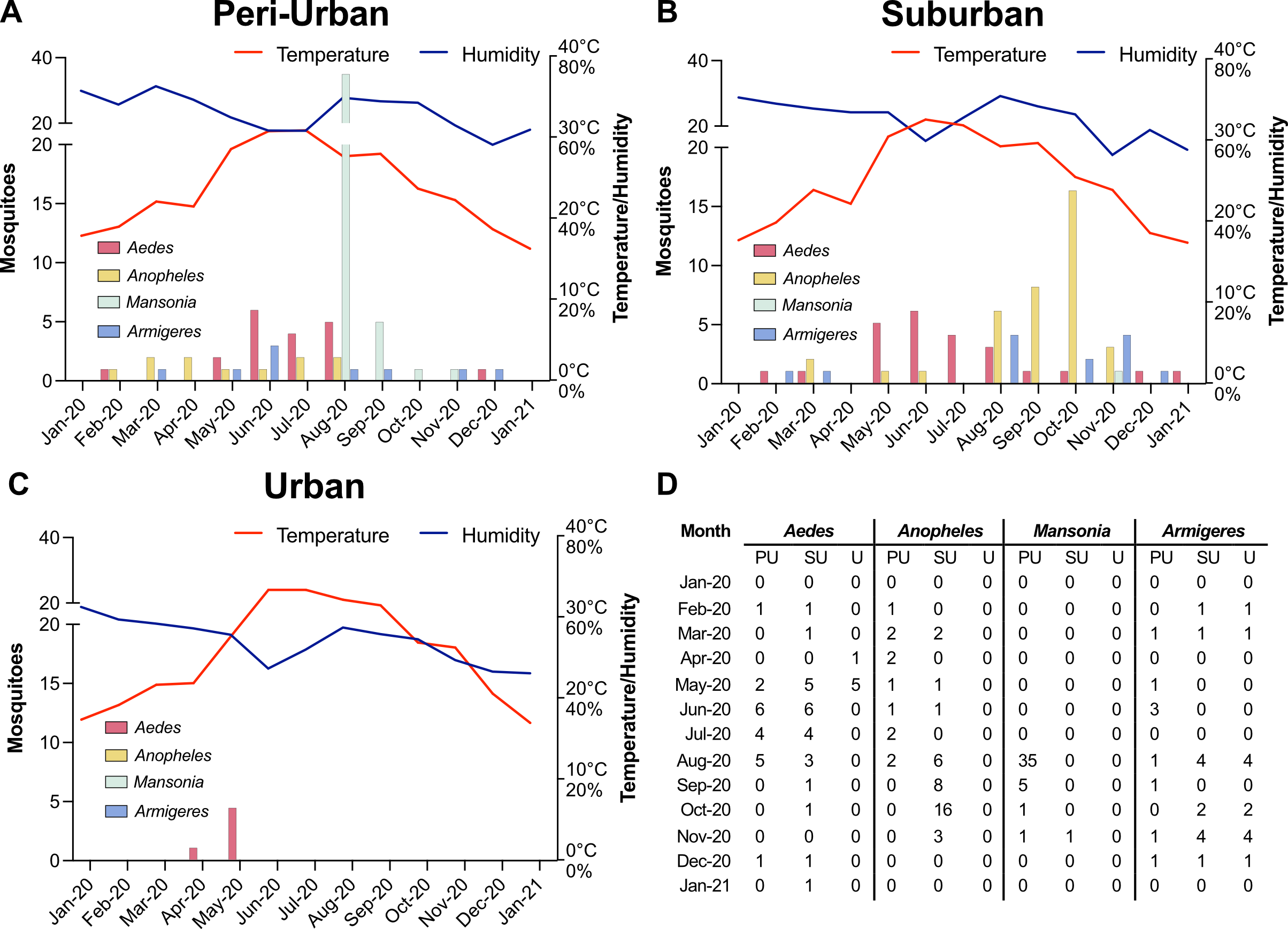

Supplement: Supplementary file 2 — Supplementary Information 2. [file 41598_2024_67438_MOESM2_ESM.tiff]
