# Supplementary material for: Study on the temporal and spatial distribution of Culex mosquitoes in Hanoi, Vietnam
Source: Sci Rep. 2024 Jul 17;14:16573. doi: 10.1038/s41598-024-67438-3 (PMC11255287; doi:10.1038/s41598-024-67438-3)
Supplement: Supplementary file 3 — Supplementary Information 3. [file 41598_2024_67438_MOESM3_ESM.docx]

| Trap | Ward | Lat | Lon |
| --- | --- | --- | --- |
| Peri-urban 1 | An Khanh | 21.008828 | 105.732711 |
| Suburban 1 | Gia Quat | 21.008828 | 105.879748 |
| Suburban 2 | Cu Khoi N | 21.0083294 | 105.9067497 |
| Suburban 3 | Cu Khoi S | 21.0081745 | 105.9067648 |
| Urban 1 | Ngoc Ha | 21.0399774 | 105.8265118 |
| Urban 2 | Kim Ma | 21.031584 | 105.818147 |
