# Supplementary material for: Study on the temporal and spatial distribution of Culex mosquitoes in Hanoi, Vietnam
Source: Sci Rep. 2024 Jul 17;14:16573. doi: 10.1038/s41598-024-67438-3 (PMC11255287; doi:10.1038/s41598-024-67438-3)
Supplement: Supplementary file 5 — Supplementary Information 5. [file 41598_2024_67438_MOESM5_ESM.docx]

| Genus | Trap location | | | | | |  |
| --- | --- | --- | --- | --- | --- | --- | --- |
| Species | An Khanh | Gia Quat | Cu Khoi N | Cu Khoi S | Ngoc Ha | Kim Ma | Total |
| *Culex* | 2346 | 1715 | 205 | 154 | 8 | 233 | 4661 |
| *Cx. fuscocephalus* | - | - | - | - | - | - | 0 |
| *Cx. gelidus* | 12 | 104 | - | - | - | - | 116 |
| *Cx. pseudovishnui* | 1 | - | - | - | - | - | 1 |
| *Cx. quinquefasciatus* | 2270 | 1489 | 200 | 146 | 8 | 231 | 4344 |
| *Cx. tritaeniorhynchus* | 56 | 122 | 5 | 8 | - | 1 | 192 |
| *Cx. vishnui* | 7 | - | - | - | - | 1 | 8 |
| *Aedes* | 19 | 17 | 5 | 2 |  | 6 | 49 |
| *Ae. aegypti* | - | - | - | - | - | 3 | 3 |
| *Ae. albopictus* | 18 | 16 | 5 | 2 | - | 3 | 44 |
| *Ae. vexans* | 1 | 1 | - | - | - | - | 2 |
| *Anopheles* | 11 | 35 | - | 2 | 1 | - | 49 |
| *Anopheles Sp.* | 11 | 35 | - | 2 | 1 | - | 49 |
| *Mansonia* | 42 | 4 | - | - | - | - | 46 |
| *Mansonia annulifera* | 35 | 2 | - | - | - | - | 37 |
| *Mansonia indiana* | 2 | 1 | - | - | - | - | 3 |
| *Mansonia Sp.* | - | 1 | - | - | - | - | 1 |
| *Mansonia uniformis* | 5 | - | - | - | - | - | 5 |
| *Armigeres* | 9 | 7 | 4 | 2 | - | - | 22 |
| *Armigeres* | 8 | 1 | 3 | 2 | - | - | 14 |
| *Armigeres kuchingensis* | 1 | 6 | 1 | - | - | - | 8 |
| *Ficalbla* | 2 | - | - | - | - | - | 2 |
| *Ficalbla minima* | 2 | - | - | - | - | - | 2 |
| Total | 2429 | 1778 | 214 | 160 | 9 | 239 | 4829 |
