# Supplementary material for: Study on the temporal and spatial distribution of Culex mosquitoes in Hanoi, Vietnam
Source: Sci Rep. 2024 Jul 17;14:16573. doi: 10.1038/s41598-024-67438-3 (PMC11255287; doi:10.1038/s41598-024-67438-3)
Supplement: Supplementary file 6 — Supplementary Information 6. [file 41598_2024_67438_MOESM6_ESM.docx]

|  | **Urban** | | **Suburban** | | **Peri-Urban** | |
| --- | --- | --- | --- | --- | --- | --- |
|  |  | |  | |  | |
|  | Temperature (C°) | Humidity (%) | Temperature (C°) | Humidity (%) | Temperature (C°) | Humidity (%) |
| Jan-20 | 22,3 | 72,4 | 18,2 | 71,6 | 18,7 | 73,1 |
| Feb-20 | 24,1 | 69,3 | 20,4 | 70,1 | 19,8 | 69,7 |
| Mar-20 | 26,6 | 68,2 | 24,4 | 68,9 | 22,9 | 74,2 |
| Apr-20 | 26,8 | 67,1 | 22,7 | 68,0 | 22,3 | 70,9 |
| May-20 | 32,6 | 65,5 | 31,0 | 68,0 | 29,4 | 66,6 |
| Jun-20 | 38,3 | 57,1 | 33,1 | 60,9 | 31,6 | 63,3 |
| Jul-20 | 38,3 | 61,8 | 32,4 | 66,6 | 31,7 | 63,3 |
| Aug-20 | 37,1 | 67,3 | 29,8 | 72,0 | 28,5 | 71,4 |
| Sep-20 | 36,4 | 65,6 | 30,2 | 69,4 | 28,8 | 70,5 |
| Oct-20 | 31,8 | 64,4 | 26,0 | 67,5 | 24,5 | 70,2 |
| Nov-20 | 31,2 | 59,2 | 24,4 | 57,5 | 23,1 | 64,6 |
| Dec-20 | 25,5 | 56,4 | 19,1 | 63,6 | 19,5 | 59,8 |
| Jan-21 | 21,9 | 56,0 | 17,9 | 58,8 | 17,1 | 63,5 |
