# Supplementary material for: Study on the temporal and spatial distribution of Culex mosquitoes in Hanoi, Vietnam
Source: Sci Rep. 2024 Jul 17;14:16573. doi: 10.1038/s41598-024-67438-3 (PMC11255287; doi:10.1038/s41598-024-67438-3)
Supplement: Supplementary file 6 [file 41598_2024_67438_MOESM4_ESM.docx]

	Trap	Number of mosquitoes	Sex	Species		Trap	Number of mosquitoes	Sex	Species		Trap	Number of mosquitoes	Sex	Species		Trap	
**Pool 1**	Peri-urban 1	13	f	Cx. quinquefasciatus	**Pool 67**	Peri-urban 1	19	f	Cx. quinquefasciatus	**Pool 124**	Suburban 1	7	f	Cx. quinquefasciatus	**Pool 181**	Peri-urban 1	
	Peri-urban 1	1	f	Cx. quinquefasciatus	**Pool 68**	Peri-urban 1	9	f	Cx. quinquefasciatus		Suburban 1	6	f	Cx. quinquefasciatus		Peri-urban 1	
	Peri-urban 1	35	f	Cx. quinquefasciatus		Peri-urban 1	1	f	Cx. quinquefasciatus		Suburban 1	8	f	Cx. quinquefasciatus	**Pool 182**	Peri-urban 1	
**Pool 2**	Peri-urban 1	50	f	Cx. quinquefasciatus		Peri-urban 1	6	f	Cx. quinquefasciatus		Suburban 1	10	f	Cx. quinquefasciatus		Peri-urban 1	
**Pool 3**	Peri-urban 1	50	f	Cx. quinquefasciatus	**Pool 69**	Peri-urban 1	2	f	Cx. quinquefasciatus	**Pool 125**	Suburban 1	2	f	Cx. tritaeniorhynchus		Peri-urban 1	
**Pool 4**	Peri-urban 1	4	f	Cx. quinquefasciatus		Peri-urban 1	10	f	Cx. quinquefasciatus	**Pool 126**	Suburban 1	2	f	Cx. quinquefasciatus	**Pool 183**	Peri-urban 1	
	Peri-urban 1	4	f	Cx. quinquefasciatus		Peri-urban 1	26	f	Cx. quinquefasciatus		Suburban 1	22	f	Cx. quinquefasciatus		Peri-urban 1	
	Peri-urban 1	6	f	Cx. quinquefasciatus		Peri-urban 1	20	f	Cx. quinquefasciatus	**Pool 127**	Suburban 1	19	f	Cx. quinquefasciatus	**Pool 184**	Peri-urban 1	
	Peri-urban 1	17	f	Cx. quinquefasciatus	**Pool 70**	Peri-urban 1	1	f	Ae. albopictus		Suburban 1	10	f	Cx. quinquefasciatus		Peri-urban 1	
**Pool 5**	Peri-urban 1	6	f	Cx. quinquefasciatus	**Pool 71**	Peri-urban 1	1	f	Ae. albopictus	**Pool 128**	Suburban 1	3	f	Cx. quinquefasciatus	**Pool 185**	Suburban 1	
	Peri-urban 1	20	f	Cx. quinquefasciatus	**Pool 72**	Peri-urban 1	1	f	Ae. albopictus		Suburban 1	2	f	Cx. quinquefasciatus		Suburban 1	
**Pool 6**	Peri-urban 1	20	f	Cx. quinquefasciatus		Peri-urban 1	2	f	Ae. albopictus		Suburban 1	4	f	Cx. quinquefasciatus		Suburban 1	
	Peri-urban 1	14	f	Cx. quinquefasciatus		Peri-urban 1	2	f	Ae. albopictus		Suburban 1	10	f	Cx. quinquefasciatus	**Pool 186**	Suburban 1	
**Pool 7**	Peri-urban 1	35	f	Cx. quinquefasciatus	**Pool 73**	Peri-urban 1	1	f	Ae. albopictus	**Pool 129**	Suburban 1	3	f	Cx. quinquefasciatus		Suburban 1	
**Pool 8**	Peri-urban 1	35	f	Cx. quinquefasciatus	**Pool 74**	Peri-urban 1	1	f	Ae. albopictus		Suburban 1	4	f	Cx. quinquefasciatus		Suburban 1	
**Pool 9**	Peri-urban 1	50	f	Cx. quinquefasciatus	**Pool 75**	Peri-urban 1	1	f	Ae. albopictus		Suburban 1	10	f	Cx. quinquefasciatus	**Pool 187**	Suburban 1	
**Pool 10**	Peri-urban 1	50	f	Cx. quinquefasciatus	**Pool 76**	Suburban 1	13	f	Cx. quinquefasciatus	**Pool 130**	Suburban 1	9	f	Cx. quinquefasciatus		Suburban 1	
**Pool 11**	Peri-urban 1	50	f	Cx. quinquefasciatus		Suburban 1	9	f	Cx. quinquefasciatus		Suburban 1	1	f	Cx. quinquefasciatus		Suburban 1	
**Pool 12**	Peri-urban 1	1	f	Cx. tritaeniorhynchus	**Pool 77**	Suburban 1	45	f	Cx. quinquefasciatus	**Pool 131**	Suburban 1	1	f	Cx. quinquefasciatus	**Pool 188**	Suburban 1	
**Pool 13**	Peri-urban 1	45	f	Cx. quinquefasciatus	**Pool 78**	Suburban 1	24	f	Cx. quinquefasciatus		Suburban 1	7	f	Cx. quinquefasciatus		Suburban 1	
**Pool 14**	Peri-urban 1	3	f	Cx. quinquefasciatus		Suburban 1	4	f	Cx. quinquefasciatus	**Pool 132**	Suburban 1	1	f	Cx. quinquefasciatus		Suburban 1	
**Pool 15**	Peri-urban 1	45	f	Cx. quinquefasciatus		Suburban 1	2	f	Cx. quinquefasciatus		Suburban 1	9	f	Cx. quinquefasciatus		Suburban 1	
**Pool 16**	Peri-urban 1	34	f	Cx. quinquefasciatus	**Pool 79**	Suburban 1	9	f	Cx. quinquefasciatus	**Pool 133**	Suburban 1	30	f	Cx. quinquefasciatus	**Pool 189**	Suburban 1	
	Peri-urban 1	4	f	Cx. quinquefasciatus		Suburban 1	5	f	Cx. quinquefasciatus		Suburban 1	10	f	Cx. quinquefasciatus	**Pool 190**	Suburban 1	
	Peri-urban 1	14	f	Cx. quinquefasciatus		Suburban 1	10	f	Cx. quinquefasciatus	**Pool 134**	Suburban 1	1	f	Ae. albopictus		Suburban 1	
**Pool 17**	Peri-urban 1	35	f	Cx. quinquefasciatus	**Pool 80**	Suburban 1	25	f	Cx. quinquefasciatus	**Pool 135**	Suburban 1	1	f	Ae. albopictus		Suburban 1	
**Pool 18**	Peri-urban 1	35	f	Cx. quinquefasciatus	**Pool 81**	Suburban 1	53	f	Cx. quinquefasciatus		Suburban 1	1	f	Ae. albopictus		Suburban 1	
**Pool 19**	Peri-urban 1	1	f	Cx. tritaeniorhynchus	**Pool 82**	Suburban 1	15	f	Cx. quinquefasciatus	**Pool 136**	Suburban 1	1	f	Ae. albopictus	**Pool 191**	Suburban 1	
**Pool 20**	Peri-urban 1	57	f	Cx. quinquefasciatus		Suburban 1	10	f	Cx. quinquefasciatus	**Pool 137**	Peri-urban 1	1	f	Cx. quinquefasciatus		Suburban 1	
**Pool 21**	Peri-urban 1	14	f	Cx. quinquefasciatus	**Pool 83**	Suburban 1	45	f	Cx. quinquefasciatus		Peri-urban 1	50	f	Cx. quinquefasciatus		Suburban 1	
	Peri-urban 1	30	f	Cx. quinquefasciatus	**Pool 84**	Suburban 1	59	f	Cx. quinquefasciatus	**Pool 138**	Suburban 1	1	f	Ae. albopictus	**Pool 192**	Suburban 1	
**Pool 22**	Peri-urban 1	35	f	Cx. quinquefasciatus	**Pool 85**	Suburban 1	16	f	Cx. quinquefasciatus		Suburban 1	1	f	Ae. albopictus		Suburban 1	
**Pool 23**	Peri-urban 1	4	f	Cx. tritaeniorhynchus		Suburban 1	15	f	Cx. quinquefasciatus		Suburban 1	1	f	Ae. albopictus	**Pool 193**	Suburban 1	
**Pool 24**	Peri-urban 1	40	f	Cx. quinquefasciatus	**Pool 86**	Suburban 1	50	f	Cx. quinquefasciatus	**Pool 139**	Suburban 1	1	f	Ae. albopictus		Suburban 1	
**Pool 25**	Peri-urban 1	40	f	Cx. quinquefasciatus	**Pool 87**	Suburban 1	71	f	Cx. quinquefasciatus	**Pool 140**	Suburban 1	1	f	Ae. albopictus	**Pool 194**	Suburban 1	
**Pool 26**	Peri-urban 1	12	f	Cx. quinquefasciatus	**Pool 88**	Suburban 1	4	f	Cx. quinquefasciatus	**Pool 141**	Peri-urban 1	1	m	Cx. quinquefasciatus	**Pool 195**	Suburban 1	
**Pool 27**	Peri-urban 1	42	f	Cx. quinquefasciatus		Suburban 1	12	f	Cx. quinquefasciatus		Peri-urban 1	2	m	Cx. quinquefasciatus		Suburban 1	
**Pool 28**	Peri-urban 1	42	f	Cx. quinquefasciatus	**Pool 89**	Suburban 1	1	f	Cx. tritaeniorhynchus		Peri-urban 1	2	m	Cx. quinquefasciatus		Suburban 1	
**Pool 29**	Peri-urban 1	40	f	Cx. quinquefasciatus		Suburban 1	1	f	Cx. tritaeniorhynchus	**Pool 142**	Peri-urban 1	12	m	Cx. quinquefasciatus		Suburban 1	
**Pool 30**	Peri-urban 1	40	f	Cx. quinquefasciatus	**Pool 90**	Suburban 1	1	f	Cx. quinquefasciatus		Peri-urban 1	2	m	Cx. quinquefasciatus	**Pool 196**	Suburban 1	
**Pool 31**	Peri-urban 1	40	f	Cx. quinquefasciatus		Suburban 1	51	f	Cx. quinquefasciatus		Peri-urban 1	8	m	Cx. quinquefasciatus	**Pool 197**	Suburban 1	
**Pool 32**	Peri-urban 1	23	f	Cx. quinquefasciatus		Suburban 1	4	f	Cx. quinquefasciatus		Peri-urban 1	3	m	Cx. quinquefasciatus		Suburban 1	
	Peri-urban 1	26	f	Cx. quinquefasciatus	**Pool 91**	Suburban 1	13	f	Cx. quinquefasciatus	**Pool 143**	Peri-urban 1	2	m	Cx. quinquefasciatus	**Pool 198**	Suburban 1	
**Pool 33**	Peri-urban 1	1	f	Cx. tritaeniorhynchus		Suburban 1	4	f	Cx. quinquefasciatus		Peri-urban 1	6	m	Cx. quinquefasciatus		Suburban 1	
**Pool 34**	Peri-urban 1	40	f	Cx. quinquefasciatus	**Pool 92**	Suburban 1	49	f	Cx. quinquefasciatus		Peri-urban 1	1	m	Cx. quinquefasciatus	**Pool 199**	Suburban 1	
**Pool 35**	Peri-urban 1	40	f	Cx. quinquefasciatus	**Pool 93**	Suburban 1	32	f	Cx. quinquefasciatus	**Pool 144**	Peri-urban 1	3	m	Cx. quinquefasciatus		Suburban 1	
**Pool 36**	Peri-urban 1	40	f	Cx. quinquefasciatus	**Pool 94**	Suburban 1	42	f	Cx. quinquefasciatus		Peri-urban 1	6	m	Cx. quinquefasciatus	**Pool 200**	Suburban 1	
**Pool 37**	Peri-urban 1	40	f	Cx. quinquefasciatus	**Pool 95**	Suburban 1	16	f	Cx. quinquefasciatus		Peri-urban 1	1	m	Cx. quinquefasciatus	**Pool 201**	Suburban 1	
**Pool 38**	Peri-urban 1	44	f	Cx. quinquefasciatus		Suburban 1	10	f	Cx. quinquefasciatus	**Pool 145**	Peri-urban 1	4	m	Cx. quinquefasciatus	**Pool 202**	Suburban 1	
**Pool 39**	Peri-urban 1	32	f	Cx. quinquefasciatus		Suburban 1	20	f	Cx. quinquefasciatus		Peri-urban 1	2	m	Cx. quinquefasciatus		Suburban 1	
**Pool 40**	Peri-urban 1	42	f	Cx. quinquefasciatus	**Pool 96**	Suburban 1	10	f	Cx. quinquefasciatus	**Pool 146**	Peri-urban 1	2	m	Cx. quinquefasciatus		Suburban 1	
	Peri-urban 1	16	f	Cx. quinquefasciatus		Suburban 1	12	f	Cx. quinquefasciatus		Peri-urban 1	2	m	Cx. quinquefasciatus	**Pool 203**	Suburban 1	
**Pool 41**	Peri-urban 1	20	f	Cx. quinquefasciatus		Suburban 1	19	f	Cx. quinquefasciatus		Peri-urban 1	1	m	Cx. quinquefasciatus	**Pool 204**	Suburban 1	
	Peri-urban 1	19	f	Cx. quinquefasciatus		Suburban 1	2	f	Cx. quinquefasciatus	**Pool 147**	Peri-urban 1	1	m	Cx. quinquefasciatus	**Pool 205**	Suburban 1	
**Pool 42**	Peri-urban 1	39	f	Cx. quinquefasciatus	**Pool 97**	Suburban 1	3	f	Cx. tritaeniorhynchus		Peri-urban 1	5	m	Cx. quinquefasciatus		Suburban 1	
**Pool 43**	Peri-urban 1	24	f	Cx. quinquefasciatus	**Pool 98**	Suburban 1	13	f	Cx. quinquefasciatus		Peri-urban 1	4	m	Cx. quinquefasciatus		Suburban 1	
	Peri-urban 1	23	f	Cx. quinquefasciatus		Suburban 1	16	f	Cx. quinquefasciatus		Peri-urban 1	5	m	Cx. quinquefasciatus	**Pool 206**	Suburban 1	
**Pool 44**	Peri-urban 1	11	f	Cx. quinquefasciatus	**Pool 99**	Suburban 1	50	f	Cx. quinquefasciatus	**Pool 148**	Peri-urban 1	4	m	Cx. quinquefasciatus		Suburban 1	
	Peri-urban 1	12	f	Cx. quinquefasciatus	**Pool 100**	Suburban 1	15	f	Cx. quinquefasciatus		Peri-urban 1	9	m	Cx. quinquefasciatus	**Pool 207**	Suburban 1	
	Peri-urban 1	5	f	Cx. quinquefasciatus	**Pool 101**	Suburban 1	2	f	Cx. tritaeniorhynchus		Peri-urban 1	4	m	Cx. quinquefasciatus		Suburban 1	
**Pool 45**	Peri-urban 1	22	f	Cx. quinquefasciatus		Suburban 1	1	f	Cx. tritaeniorhynchus		Peri-urban 1	12	m	Cx. quinquefasciatus	**Pool 208**	Suburban 1	
	Peri-urban 1	10	f	Cx. quinquefasciatus		Suburban 1	1	f	Cx. tritaeniorhynchus	**Pool 149**	Peri-urban 1	10	m	Cx. quinquefasciatus	**Pool 209**	Suburban 1	
